# 116 Introduction of a Procalcitonin Screening Protocol in Severe Burn Patients: A Quality Improvement Initiative

**DOI:** 10.1093/jbcr/irae036.115

**Published:** 2024-04-17

**Authors:** Meghna Kurup, Lauren B Nosanov, Jack Bullis, Lori Mickelson, Amanda Meyer, Kristin E Friedl, Cindy L Schmitz, Angela Gibson, Lee D Faucher

**Affiliations:** University of Wisconsin, Madison, WI; University of Wisconsin, Middleton, WI; University of Wisconsin (UW Health), Madison, WI; University of Wisconsin Madison, Middleton, Wisconsin; University of Wisconsin Burn Center, Stoughton, Wisconsin; UW Health, Waunakee, Wisconsin; University of Wisconsin School of Medicine and Public Health, Madison, WI; University of Wisconsin, Madison, WI; University of Wisconsin, Middleton, WI; University of Wisconsin (UW Health), Madison, WI; University of Wisconsin Madison, Middleton, Wisconsin; University of Wisconsin Burn Center, Stoughton, Wisconsin; UW Health, Waunakee, Wisconsin; University of Wisconsin School of Medicine and Public Health, Madison, WI; University of Wisconsin, Madison, WI; University of Wisconsin, Middleton, WI; University of Wisconsin (UW Health), Madison, WI; University of Wisconsin Madison, Middleton, Wisconsin; University of Wisconsin Burn Center, Stoughton, Wisconsin; UW Health, Waunakee, Wisconsin; University of Wisconsin School of Medicine and Public Health, Madison, WI; University of Wisconsin, Madison, WI; University of Wisconsin, Middleton, WI; University of Wisconsin (UW Health), Madison, WI; University of Wisconsin Madison, Middleton, Wisconsin; University of Wisconsin Burn Center, Stoughton, Wisconsin; UW Health, Waunakee, Wisconsin; University of Wisconsin School of Medicine and Public Health, Madison, WI; University of Wisconsin, Madison, WI; University of Wisconsin, Middleton, WI; University of Wisconsin (UW Health), Madison, WI; University of Wisconsin Madison, Middleton, Wisconsin; University of Wisconsin Burn Center, Stoughton, Wisconsin; UW Health, Waunakee, Wisconsin; University of Wisconsin School of Medicine and Public Health, Madison, WI; University of Wisconsin, Madison, WI; University of Wisconsin, Middleton, WI; University of Wisconsin (UW Health), Madison, WI; University of Wisconsin Madison, Middleton, Wisconsin; University of Wisconsin Burn Center, Stoughton, Wisconsin; UW Health, Waunakee, Wisconsin; University of Wisconsin School of Medicine and Public Health, Madison, WI; University of Wisconsin, Madison, WI; University of Wisconsin, Middleton, WI; University of Wisconsin (UW Health), Madison, WI; University of Wisconsin Madison, Middleton, Wisconsin; University of Wisconsin Burn Center, Stoughton, Wisconsin; UW Health, Waunakee, Wisconsin; University of Wisconsin School of Medicine and Public Health, Madison, WI; University of Wisconsin, Madison, WI; University of Wisconsin, Middleton, WI; University of Wisconsin (UW Health), Madison, WI; University of Wisconsin Madison, Middleton, Wisconsin; University of Wisconsin Burn Center, Stoughton, Wisconsin; UW Health, Waunakee, Wisconsin; University of Wisconsin School of Medicine and Public Health, Madison, WI; University of Wisconsin, Madison, WI; University of Wisconsin, Middleton, WI; University of Wisconsin (UW Health), Madison, WI; University of Wisconsin Madison, Middleton, Wisconsin; University of Wisconsin Burn Center, Stoughton, Wisconsin; UW Health, Waunakee, Wisconsin; University of Wisconsin School of Medicine and Public Health, Madison, WI

## Abstract

**Introduction:**

Sepsis is a major cause of mortality in critically ill burn patients, in whom lab findings and signs can be non-specific for infection, and definitive microbial identification can take 2-4 days, potentially leading to treatment delays and poor antimicrobial stewardship. Adjunct tests could expedite sepsis detection and aid in treatment decisions. Existing literature suggests a role for serum procalcitonin (PCT) as a useful biomarker for early diagnosis of sepsis in burn patients. However, no consensus exists on the frequency of testing or clinically relevant thresholds and how they should guide medical decisions. This QI project set out to assess Burn Center provider perceptions on the utility of PCT screening through protocol development and implementation.

**Methods:**

The institutional registry was queried for all patients admitted to the Burn service (inclusive of non-burn etiologies) 5/2023-8/2023 following introduction of a twice weekly PCT screening protocol. Screening was to start at admission or when clinically indicated and cease at time of discharge or total wound closure. The triggers for screening include: adults with > 20% total body surface area (TBSA) or > 10% TBSA with immunocompromise (including significant alcohol abuse and uncontrolled diabetes), hospitalization > 30 days, inhalation injury, and adults or children with any TBSA at attending discretion. Retrospective chart review gathered data on demographics, injury characteristics, infectious workups, and clinical outcomes. Providers were queried as to the utility they felt PCT had played in their patient management during this timeframe.

**Results:**

A total of 112 patients (62 adult, 50 pediatric) were identified in the three-month study period, with 19.4% of adults meeting one or more triggers. PCT levels were drawn in all who met triggers except for one moribund patient who rapidly transitioned to comfort measures. Antibiotics outside of the perioperative period were given in 11.3%, and 8.1% had fever workups performed. In the pediatric population PCT was not checked during the index hospitalization -- 8.0% received antibiotics, but none required work-up for fever of unknown source. At the time of study conclusion Burn team members were equivocal regarding PCT screening and opted to continue assessing the protocol for an additional three months.

**Conclusions:**

Our chosen triggers seemed appropriate to prompt patient screening. However, critically ill patients co-managed with the SICU were not reliably captured as it required another team with less protocol familiarity to order labs. Future work will entail refining the PCT screening protocol to help determine testing frequency, useful cut-offs, and algorithms for decision making.

**Applicability of Research to Practice:**

Determining the utility of PCT screening in aiding the detection of sepsis could help reduce mortality-increasing treatment delays in severe burn patients.

